# Gender Effect on Clinical Profiles, Pharmacological Treatments and Prognosis in Patients Hospitalized for Heart Failure

**DOI:** 10.3390/jcm13030881

**Published:** 2024-02-02

**Authors:** Luca Fazzini, Mattia Casati, Alessandro Martis, Ferdinando Perra, Paolo Rubiolo, Martino Deidda, Giuseppe Mercuro, Christian Cadeddu Dessalvi

**Affiliations:** 1 Department of Medical Sciences and Public Health, University of Cagliari, 09124 Cagliari, Italy; luca.fazzini10@gmail.com (L.F.); mattiacasati1996@gmail.com (M.C.); amartismed@gmail.com (A.M.); ferdinando.perra@gmail.com (F.P.); paolo.f.rubiolo@gmail.com (P.R.); giuseppemercuro@gmail.com (G.M.); cadedduc@unica.it (C.C.D.); 2 Sassu Cardiologic Center, Cittadella Universitaria, 09033 Sardinia, Italy

**Keywords:** acute heart failure, gender medicine, pharmacological treatments, prognosis

## Abstract

Heart failure (HF) is a significant disease affecting 1–2% of the general population. Despite its general aspects, HF, like other cardiovascular diseases, presents various gender-specific aspects in terms of etiology, hemodynamics, clinical characteristics, therapy, and outcomes. As is well known, HF with preserved ejection fraction more frequently affects females, with diabetes and arterial hypertension representing the most critical determinants of HF. On the other hand, women are traditionally underrepresented in clinical trials and are often considered undertreated. However, it is not clear whether such differences reflect cultural behaviors and clinical inertia or if they indicate different clinical profiles and the impact of sex on hard clinical outcomes. We aimed to review the sex-related differences in patients affected by HF.

## 1. Introduction

Heart failure (HF) is a prevalent and progressive clinical syndrome characterized by cardinal symptoms and typical signs. It arises from structural and functional abnormalities in the heart, leading to elevated intracardiac pressures or inadequate cardiac output, both at rest and during exercise.

The classification of HF has been delineated into distinct phenotypes based on left ventricular ejection fraction (LVEF): HF with reduced EF (HFrEF) with an EF ≤ 40%, HF with mildly reduced fraction (HFmrEF) with an EF ranging from 41% to 49%, and HF with preserved EF (HFpEF) with an EF greater than 50% [1].

Noteworthy gender disparities exist within the HF spectrum. HFrEF predominantly affects men, while women are more predisposed to HFpEF due to distinct comorbidities such as hypertensive heart disease (more prevalent in females) and diabetes. Additionally, sex-specific pathophysiological factors, including pregnancy-related disorders, nulliparity, loss of estrogen, premature menopause, and consequences of breast cancer treatments such as chemotherapy and radiotherapy-induced cardiomyopathy, contribute to these differences [1,2,3,4].

## 2. Gender Differences in Risk Factors for Heart Failure

There is a growing body of evidence highlighting the significance of gender differences in the epidemiology, pathophysiology, treatment, and outcomes of various diseases, and cardiovascular disease (CVD) is no exception. Sex differences refer to biology-related distinctions between women and men, stemming from diverse sex chromosomes, sex-specific gene expressions of autosomes, sex hormones, and their respective impacts on organ systems. Concurrently, gender differences result from sociocultural processes, encompassing distinct behaviors, exposure to specific environmental influences, dietary patterns, lifestyles, stress, and variations in attitudes toward treatments and prevention between women and men [5].

Furthermore, women face sex-specific risk factors for HF, particularly associated with complications arising from pregnancy [4].

**Figure 1:**
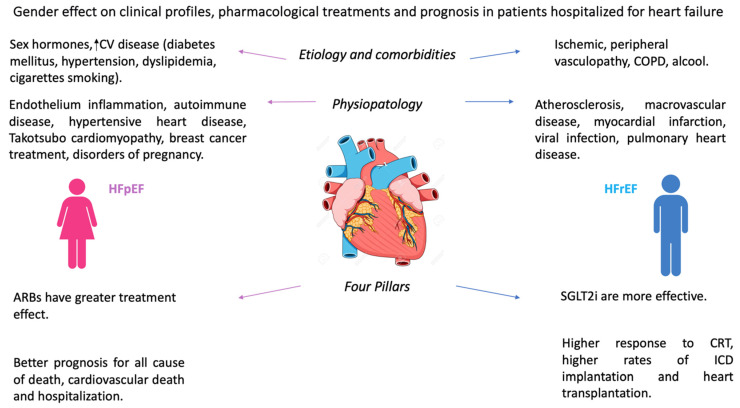
Main gender differences in clinical profile, pharmacological treatment, and prognosis in patients hospitalized for heart failure. COPD: chronic obstructive pulmonary disease. ARBs: angiotensin receptor blockers. SGLT2i: sodium-glucose co-transporter 2 inhibitor. CRT: cardiac resynchronization therapy. ICD: implantable cardioverter defibrillator.

Epidemiological studies indicate that diabetes mellitus (DM) presents a more potent risk factor for CVD in women compared to men [6]. As reported by Kautzky-Willer et al., sex hormones play a significant role in influencing energy metabolism, body composition, vascular function, and inflammatory responses [7]. Indeed, endocrine imbalances are associated with unfavorable cardiometabolic traits, evident in women with androgen excess or men with hypogonadism [7]. While in men, DM is often diagnosed at a younger age and lower body mass index (BMI), obesity, a prominent risk factor, is more prevalent in women [7] (Figure 1).

Therefore, crucial parameters to consider include BMI, with normal values ranging from 18.5 kg/m^2^ to 24.99 kg/m^2^, and waist circumference, with reference values of <80 cm in women and <94 cm in men. The Framingham study showed that women with obesity have an increased risk of coronary heart disease (CHD) of 64% compared to 46% among men with obesity [8].

Furthermore, in the realm of pregnancy-related risk factors for HF, conditions such as gestational diabetes and preeclampsia are at the forefront. The INTERHEART study pointed out that gestational diabetes heightens the likelihood of developing type 2 diabetes and experiencing a myocardial infarction [9]. Similarly, preeclampsia increases the risk of hypertension, coronary artery disease, stroke, and HF for up to four decades following the pregnancy.

Evidence suggests that the evolution of lifetime blood pressure (BP) varies between women and men, potentially leading to an increased CVD risk at lower BP thresholds [5]. Generally, the diagnosis and treatment of hypertension are similar between genders, except for women of childbearing potential or during pregnancy. The 2023 ESC Guidelines for the management of cardiovascular disease in patients with diabetes emphasize how during these periods, certain drugs, such as RAS blockers, can have adverse effects on the fetus, particularly in early gestation [6].

These distinct risk factors are not isolated pathologies but rather interact with each other. When compared to women and men without DM, women typically exhibit more notable differences in BP and higher rates of hypertension than men at the time of DM diagnosis [10]. Additionally, women tend to have poorer BP control following diagnosis. Furthermore, sex-specific hypertension-mediated organ damage is associated with a significantly elevated risk of HF with preserved ejection fraction (HFpEF) in women, especially in the presence of DM [6]. Ventura-Clapier et al. demonstrated that women with hypertension have a 3-fold higher risk of HF or stroke than men and have higher rates of recurring myocardial infarction (MI) after an initial MI [11].

Evidence indicates that sex hormones and sex-specific molecular mechanisms play a role in influencing glucose and lipid metabolism, as well as cardiac energy metabolism and function. Males tend to have a more pro-atherogenic lipid profile, characterized by lower high-density lipoprotein and higher low-density lipoprotein and triglycerides [12].

Dyslipidemia emerges as a significant contributor to gender-based variations observed in HF. As shown by Meloni A. et al., the impact of abnormal lipid profiles, including elevated levels of cholesterol and triglycerides, varies between men and women, influencing the development and progression of HF differently [13]. Recognizing these gender-specific aspects of dyslipidemia is crucial for tailoring effective preventive and therapeutic strategies for HF in both male and female populations.

Cigarette smoking accounts for 50% of all preventable deaths in smokers, with half of these attributable to atherosclerotic cardiovascular disease. Notably, prolonged smoking poses a greater risk for women than men [5]; however, a meta-analysis of over three million individuals demonstrated that except for women aged 30–44 years, female smokers had a 25% greater risk of CVD than male smokers [14].

Young women who smoke face an elevated risk of sudden death due to MI, the pathology most strongly associated with smoking. The risk of myocardial infarction in male smokers is approximately five times higher than in women, with an increase corresponding to the number of cigarettes smoked. This difference is believed to be linked to the protective role of female hormones in the cardiovascular system [15].

In summary, given the under-representation of women in clinical trials and the absence of evidence for sex-specific recommendations regarding CVD management, the implementation of sex-balanced recruitment strategies is recommended for future cardiovascular outcome trials. Most importantly, concerted efforts should be made to ensure that women receive equal healthcare opportunities in the management of CVD.

## 3. Gender Differences in Pathophysiology

As remarked by Lam et al., microvascular dysfunction is attributed to endothelial inflammation, often stemming from cardiometabolic comorbidities such as obesity, which is more prevalent in females, and diabetes, disrupting the nitric oxide (NO) pathway [1].

In women, many sex-related conditions can lead to microvascular disease, such as postmenopausal estrogen loss and a higher tendency of autoimmune diseases, consequently leading to the increased production of pro-inflammatory cytokines. This, coupled with diastolic dysfunctions often caused by autoimmune diseases, adds to the complexity of the cardiovascular scenario [1].

Ischemic cardiopathy is the primary cause of HF with reduced ejection fraction (HFrEF) in both genders, while among the non-ischemic causes, hypertensive heart disease emerges as the predominant cause of HF with preserved ejection fraction (HFpEF). Long-standing hypertensive heart disease can progress to HF through cardiomyocyte dysfunction, fibrosis due to increased extracellular matrix, and the rarefaction of intramyocardial microvasculature. Diastolic dysfunction commonly presents early in HFpEF caused by hypertensive heart disease, induced by persistent pressure overload, and it results in concentric left ventricular hypertrophy [16].

An acute and transient HF presentation is frequently observed in Takotsubo cardiomyopathy, being more prevalent in females and triggered by impaired neurohormonal regulation during acute emotional or psychological stress. Women experiencing higher psychological distress are more prone to developing cardiovascular events than men [1]. As described in Circulation by Pelliccia et al., about 90% of patients with Takotsubo syndrome are postmenopausal women; women are also more predisposed to experience Takotsubo major adverse events, including cardiogenic shock, cardiac arrest, and mortality [17].

Breast cancer, the most prevalent cancer in females, presents an additional risk of HF due to cardiotoxicity from modern anti-cancer treatments, including chemotherapy and radiotherapy. Cadeddu Dessalvi et al., reported in their review that anthracyclines, which are a cornerstone for breast cancer and many other oncologic treatments, have a higher cardiotoxicity in the female sex, and this may be explained by gender differences in metabolic pathways which represent an intriguing ongoing research field to obtain more tailored therapies [18,19]. Radiotherapy, on the other hand, is associated with a heightened risk of major coronary events, such as myocardial infarction and coronary revascularization. This risk increases linearly with the mean dose to the heart, starting within the first 5 years post-radiation and continuing for at least 20 years. While cardiomyocytes are resistant to radiation, radiotherapy induces microvascular endothelial damage, leading to coronary microvascular artery rarefaction, oxidative stress, and fibrosis. Breast cancer and CVD share common risk factors, including age, obesity, and tobacco use, which may contribute to HF development [20,21].

## 4. Gender Differences in Diagnostic and Clinical Presentation

Diagnostic and clinical approaches at the onset of HF may vary based on the HF phenotype, clinical presentation, and gender. In terms of clinical presentation, reduced ejection fraction (EF) is more often associated with the male sex, whereas HFpEF is more prevalent in females and is linked to worse clinical outcomes [22,23]. At the time of diagnosis, women tend to present symptoms of chronic HF, such as exertional dyspnea, jugular vein distention, and peripheral edema, more frequently than men [22,23,24].

HF is primarily diagnosed clinically, stemming from structural and/or functional cardiac abnormalities. While the diagnosis is mainly clinical, confirmation and phenotype classification require an echocardiographic study; alternative diagnostic tools may be advantageous in specific scenarios [25]. Diagnostic tools that expose patients to radiation, such as computed tomography and nuclear imaging, are less frequently requested in females [25].

Biomarker plasma concentrations play a pivotal role in HF diagnosis, and the difference between men and women can only be partially explained by hormone status. Given the higher prevalence of HFpEF in women, natriuretic peptides are lower compared to men, as men are more affected by HFrEF, which typically carries higher natriuretic peptide levels. There is substantial evidence that most biomarkers, regardless of gender, exhibit similar diagnostic and prognostic effectiveness [26].

When an ischemic etiology is suspected, patients undergo coronary angiography without gender differences, but women are less likely to undergo percutaneous coronary intervention (PCI) in one-vessel disease. They are more likely to undergo PCI in multi-vessel disease and less likely to undergo coronary artery bypass graft (CABG) [27]. Diagnostic imaging is crucial in detecting ischemic etiology, particularly in women who frequently present ischemia and vascular dysfunction without obstructive coronary artery disease or due to spontaneous coronary artery dissection [28]. Despite the proven efficacy of pharmacological therapy, intracoronary imaging, and revascularization, women undergo invasive and non-invasive interventional strategies less frequently than men [29].

Regarding dilated cardiomyopathy, no significant gender differences exist in diagnosis, and the relationship between gender and the expression of pathogenic gene mutations remains unclear. Therefore, the role of genetic testing is similar between genders. However, a gender difference is noted in alcoholic cardiomyopathy, more common in men due to higher alcohol consumption [30]. Among secondary dilated cardiomyopathies, peripartum cardiomyopathy affects females and should be considered in the differential diagnosis for female patients presenting with dyspnea during pregnancy or postpartum [31].

Stress cardiomyopathy is more frequent in the female sex, with men commonly presenting with a physical trigger and being more prone to developing cardiogenic shock with worse clinical outcomes [32]. Myocarditis incidence is not significantly dissimilar between genders, but some registries indicate that men are hospitalized more frequently than women, despite there being higher mortality rates in women [33].

## 5. Gender Differences in Medical Treatment and Relationship with Invasive Cardiological Care or General Medicine Care

### 5.1. Medical Treatment

Current guidelines do not differentiate HF therapies between women and men, despite evidence pointing to gender differences. Primarily, variations in pharmacokinetics and pharmacodynamics contribute to differences in drug absorption, distribution, metabolism, excretion, and, consequently, drug effects [34,35].

Moreover, adverse drug effects vary between genders, with women experiencing 1.5 times higher rates than men. For instance, women with HF receiving diuretic therapy are more prone to ion imbalance and subsequent severe arrhythmias or ACE inhibitor cough [36].

The benefits of beta-blocker treatment in the context of HF with reduced ejection fraction are well-established for both males and females, as demonstrated by the “COPERNICUS” and “CIBIS II” trials [37,38]. Although the “MERIT-HF” trial did not find a beneficial effect on mortality in small subgroups of women [39,40], a post hoc analysis revealed a reduction in all-cause death or hospitalization in both women and men, with a more marked difference in the reduction in the risk of HF hospitalization in women [41].

In all the mentioned trials, females have been notably underrepresented, including in the evaluation of the efficacy of ACE inhibitors and angiotensin receptor blockers (ARBs) in HF patients. A post hoc analysis of the “CONSENSUS” study did not show a significant reduction in the primary endpoint of death with the use of Enalapril in women, as observed in men [37,42,43]. Meanwhile, the ATLAS and HEAAL trials suggested that lower doses of Lisinopril and Losartan may be effective in women, while men may require higher doses [44,45].

ARBs may have a more significant treatment effect in females than males in HFpEF. In the I-PRESERVE trial, Irbesartan showed a lower rate of all-cause mortality or first cardiovascular hospitalization in women compared to the male subgroup [46].

One of the new milestones in HFrEF treatment is the angiotensin receptor neprilysin inhibitors (ARNi). The PARADIGM-HF trial significantly favored sacubitril/valsartan over Enalapril in both males and females for the composite endpoint of cardiovascular mortality and HF hospitalization, with no significant sex differences [47].

In HFpEF patients, ARNi showed a significant effect on the composite endpoint only in the female subgroup (RR 0.73 vs. 1.03 in males), with females appearing more responsive to treatment at higher LVEF ranges than men [48,49]. In a subgroup analysis of the PROVE-HF trial, the initiation of sacubitril/valsartan in women demonstrated more rapid reductions in NT-proBNP and earlier reverse left ventricular remodeling [50].

As the RALES and EMPHASIS-HF trials reveal, using mineralocorticoid receptor antagonists is associated with reducing all-cause death in both males and females in the NYHA III-IV class, with no sex differences [51,52]. However, there is a sex disparity among HFpEF patients, as the TOPCAT trial demonstrates that spironolactone reduces the risk of all-cause death in females but not in males (HR 0.66 vs. 1.06) [53].

The last pillar in the treatment of HFrEF is represented by sodium–glucose cotransporter-2 inhibitors such as Dapagliflozin and Empagliflozin. Both drugs appear to provide benefits, such as a reduction in cardiovascular death and HF hospitalization, in both genders without significant gender disparity, as revealed in the DAPA-HF and EMPEROR-Reduced trials [54,55]. The newest EMPEROR-Preserved trial describes similar effects of Empagliflozin treatment in patients with HFpEF [56]. At the same time, a systematic review and meta-analysis of the five most essential trials about SGLT-2i, including DELIVER and SOLOIST, define that the reduction in worsening HF and death from cardiovascular causes was less pronounced in women [57].

In HFrEF patients, the use of digoxin determines the reduction in HF-related hospitalizations, as shown by the DIG trial, but a post hoc analysis defined a higher risk of all-cause mortality in women compared to men [58].

### 5.2. Invasive Cardiological Care

In the context of ischemic cardiomyopathy, there is a sex-specific disparity in access to coronary artery bypass grafting (CABG) [59]. However, over ten years, women exhibited lower all-cause mortality and cardiovascular mortality than men, emphasizing the critical importance of avoiding any delays in surgery based on gender [60].

Furthermore, gender differences come into play in the management of secondary mitral regurgitation (SMR) resulting from left ventricular remodeling in HFrEF patients. Women experience delayed referral for surgical intervention, leading to a less favorable scenario at presentation, fewer opportunities for valve repair, and a worse postoperative prognosis [61,62]. The quantitative cutoff values for effective regurgitant orifice area (EROA) and regurgitant volume are not adjusted for gender, potentially contributing to an overestimation of SMR severity in women [63,64].

A sub-analysis of the COAPT trial revealed that women undergoing transcatheter mitral valve repair with the MitraClip had a worse quality of life and functional capacity compared to men. Although transcatheter edge-to-edge repair (TEER) resulted in improved outcomes for both genders, the benefits were less pronounced in females (HR 0.78 vs. 0.43 in men) [65].

## 6. Gender Differences in Non-Medical Treatment: Devices and Surgery

### 6.1. Cardiac Resynchronization Therapy

Cardiac resynchronization therapy (CRT-P and -D) is now well established as a therapeutic option for selected patients with HFrEF and a prolonged QRS interval or when a high threshold of right ventricle pacing is expected [66]. CRT significantly improves HF symptoms, reduces hospitalizations, and lowers mortality [67,68,69,70,71].

It is essential to note that women are underrepresented in CRT trials, comprising only about 20% of enrollees, which complicates the assessment of sex differences. However, females tend to derive more significant benefits from CRT, particularly in terms of reductions in mortality, compared to males. Despite this, women undergo device implantation less frequently [72]. Furthermore, females exhibit a high response rate irrespective of QRS duration, experiencing a decrease in mortality and HF hospitalization for QRS durations between 130 and 149 ms in CRT-D recipients. This highlights that a 150 ms duration threshold for CRT implantation might be a limiting factor in accessing therapy [12].

Fewer women than men undergo CRT implantation [68,69], and a relatively higher percentage of these patients receive CRT-P instead of CRT-D in Europe [73], with reasons for this choice remaining unclear. The net clinical benefit from CRT seems similar between genders, although some evidence suggests that response rates in women may be superior to those in men [68,69,74,75]. This is possibly linked to a lower rate of ischemic etiology and fewer scarred segments at baseline compared to men [76]. In a meta-analysis by Zusterzeel et al. [77], it was found that women exhibit up to a 76% risk reduction compared to men, suggesting the need for a sex-specific definition of left bundle branch block for patient selection in CRT, with a potentially lower QRS duration cut-off value for women and men [1]. Conversely, a recent study revealed that this sex difference may not be a sex-specific result but may rather be because the smaller height and heart size of women are the actual predictors of being a responder to CRT [78].

### 6.2. Implantable Cardioverter Defibrillator

The implantable cardioverter defibrillator has demonstrated efficacy in reducing sudden cardiac death in both primary and secondary prevention. However, a notable challenge is the underrepresentation of women in randomized control trials evaluating ICD therapy, constituting only 10–32% of enrolled patients [79]. This insufficient representation hampers the ability of trials to adequately assess sex-specific outcomes. Despite this, findings from CRT trials indicate that there is not a significant interaction by sex regarding the benefits of ICD therapy, irrespective of whether the cardiomyopathy is ischemic or non-ischemic [74,80,81,82].

Aggregate registry data reveal that woman receiving an ICD experience lower mortality and a reduced incidence of proper therapies for life-threatening arrhythmias. However, it is important to note that they also face higher rates of complications, including infection and pneumothorax [83]. Additionally, women inherently exhibit a lower lifetime risk of ventricular arrhythmias and sudden cardiac death compared to men.

While various devices such as Cardiac Contractility Modulation and Cardio-MEM are now available, there is currently no robust evidence indicating significant gender differences in their effectiveness. Further research is needed to elucidate the potential nuances in outcomes across genders associated with these emerging technologies.

### 6.3. Heart Transplantation

Heart transplantation (HT) remains the gold standard for treating advanced HF, yet a significant gender disparity exists, with women constituting only approximately 25% of heart transplant recipients annually. This is in contrast to real-world population studies, which suggest that women make up to 45% of individuals with advanced HF [84]. Research by DeFilippis et al. highlights that women are less likely to be referred for HT and left ventricular assist devices (LVADs), despite their higher incidence of HF [85]. Moreover, women face higher waitlist mortality during the HT evaluation process and encounter more allo-sensitization disadvantages compared to men. It is noteworthy that women listed for HT are generally younger than their male counterparts and exhibit a distinct distribution of HF etiology [86].

Despite these disparities, early and late mortality outcomes after HT do not show significant differences between genders [87] (Table 1). However, during the follow-up period, women experience higher rates and a greater severity of rejection but demonstrate a lower prevalence of cardiac allograft vasculopathy and lower rates of malignancies compared to men [85]. These nuances highlight the need for a more comprehensive understanding of gender-specific factors influencing the entire heart transplantation process, from referral to post-transplant outcomes.

### 6.4. Left Ventricular Assist Devices and Surgery

Recent data derived from INTERMACS reveal that females constitute approximately 21.4–22.7% of total LVAD implantations [88]. The Momentum-3 trial, however, did not observe a significant interaction between gender groups in their prespecified subgroup analysis [89]. Conversely, an observational study focusing on LVAD recipients suggests that women face a higher risk of mortality, reduced likelihood of heart transplantation, and an increased rate of adverse events [90]. These disparities in clinical outcomes persist even when stratified by race, device strategy, or implantation center (Table 1).

In the realm of surgical interventions for patients with HFrEF, CABG is a common procedure. This may be performed alone or combined with surgical ventricular reconstruction and mitral valve surgery for regurgitation. Limited data from randomized trials indicate that sex is not a significant factor associated with the effects of CABG plus medical therapy compared to medical therapy alone, specifically concerning all-cause mortality and cardiovascular mortality. Consequently, treatment decisions about CABG in these patients should not be influenced by gender considerations [91].

## 7. Overall Prognosis and Therapy Limitations in Both Genders

The prognostic stratification of HF poses a clinical challenge, particularly in women. HF in women exhibits specific characteristics in clinical presentation, response to therapy, and adherence to guidelines, leading to disparities between men and women [92,93,94] (Figure 1). Moreover, the underrepresentation of women in randomized clinical trials contributes to a lack of sex-oriented assessment in current prognostic scores [92].

Numerous studies indicate that women with HF generally experience better survival rates and lower hospitalization rates than their male counterparts. In the Olmsted County study, age-adjusted all-cause mortality rates were comparable between genders, while cardiovascular death rates were higher in men, and hospitalization rates were lower in women [95,96]. This pattern could be attributed to a lower prevalence of ischemic myocardial disease and a later onset of symptoms in older women [46,92]. Notably, the most common phenotype of HF in women is non-ischemic HFpEF [1]. In the acute HF setting, the ARIC study reported similar 28-day and 1-year case fatality rates between men and women (10% and 30%, respectively) [97].

Furthermore, the impact of chronic HF on quality of life appears to be more pronounced in women than in men [98]. Women affected by HF report more significant physical limitations and higher rates of anxiety and depression than their male counterparts, potentially influencing the effectiveness of therapy [24,99]. Additionally, as women are more frequently affected by HFpEF and often present with comorbidities such as chronic kidney disease, their ability to receive a complete prescription of optimal medical therapy may be limited, impacting prognosis [100].

## 8. Future Perspectives

Heart failure represents one of the most critical challenges for cardiology. While the development of highly effective diagnostic and therapeutic strategies has improved the survival and quality of life of patients with cardiovascular pathologies, it has concurrently expanded the population affected by heart failure.

In this context, there is a pressing need for an increased focus on gender-specific characteristics in pathophysiology, pharmacokinetics, and pharmacodynamics. The ongoing efforts of researchers in the realm of personalized medicine must systematically incorporate gender as a pivotal factor amidst biological, environmental, behavioral, and psychological considerations.

Moreover, given the current scarcity of robust gender-oriented data in the scientific literature, substantial attention should be directed toward the design of basic research and randomized clinical trials. These endeavors aim to elucidate gender-related distinctions in both disease manifestation and therapeutic responses, contributing to a more comprehensive understanding of heart failure and paving the way for tailored and effective interventions.

## 9. Conclusions

In this review, we have highlighted notable gender differences in the context of HF, emphasizing that men are more susceptible to HFrEF, while women are predisposed to HFpEF due to distinct comorbidities. We have delved into the variations in risk factors, pathophysiology, and clinical presentation, noting that women with HF generally exhibit better survival rates. However, the impact of the disease on their quality of life can be more substantial.

A significant concern that persists is the underrepresentation of women in clinical trials related to HF. This disparity raises questions about the applicability of research findings to women, highlighting the need for a more inclusive approach in clinical studies. Additionally, there is an urgent requirement for healthcare professionals to integrate sex-specific considerations into the diagnosis, treatment, and hospital care of individuals with HF, ensuring that both men and women receive optimal and tailored interventions. This ongoing issue underscores the importance of addressing gender disparities to enhance overall management and outcomes in HF.

## Figures and Tables

**Table 1:** Gender differences in CABG, MitraClip, heart transplant, and LVAD according to reported outcomes. CABG: coronary-artery bypass grafting. CAV: cardiac allograft vasculopathy. LVAD: left ventricular assist device.

Therapy	Endpoint	Male	Female
CABG	Mortality	↑	↓
MitraClip	Survival	↑	↓
	Quality of life	↑	↓
Heart Transplant	% of patients	≈75%	≈25%
	Possibility of referral	↑	↓
	Waiting list mortality	↓	↑
	Early and late mortality	=	=
	Rejection	↓	↑
	CAV	↓	↑
	Malignancies	↓	↓↑=
LVAD	% of patients	≈78%	≈22%
	Mortality	↓	↑
	Bridge to transplant	↑	↓
	Adverse events	↓	↑
